# PHLPP1 deletion restores pancreatic β-cell survival and normoglycemia in the db/db mouse model of obesity-associated diabetes

**DOI:** 10.1038/s41420-022-00853-5

**Published:** 2022-02-08

**Authors:** Blaz Lupse, Nick Heise, Kathrin Maedler, Amin Ardestani

**Affiliations:** 1 Centre for Biomolecular Interactions Bremen, University of Bremen, Bremen, Germany; 2 Department of Molecular Medicine, School of Advanced Technologies in Medicine, Tehran University of Medical Sciences, Tehran, Iran

**Keywords:** Type 2 diabetes, Apoptosis

## Abstract

The Pleckstrin homology domain leucine-rich repeat protein phosphatases (PHLPPs) are novel therapeutic targets for the restoration of β-cell survival and function in diabetes. Their upregulation and activation in β-cells under conditions of both type 1 and type 2 diabetes directly correlates with β-cell failure; β-cell death and loss of insulin secretory function through disturbance of cell survival control mechanisms. PHLPPs directly dephosphorylate and regulate activities of β-cell survival-dependent kinases AKT and MST1 constituting a regulatory triangle loop to control β-cell apoptosis. PHLPP1 deletion in severely diabetic leptin receptor-deficient db/db mice restored normoglycemia and β-cell area through increased β-cell proliferation and reduced β-cell apoptosis. The beneficial effects of PHLPP1 deficiency in a severe mouse model of obesity and diabetes make PHLPP a new target for β-cell-directed diabetes therapy.

Type 2 diabetes (T2D) is a sophisticated metabolic disease defined by systemic insulin resistance as well as a decline in functional pancreatic β-cell mass. A progressive β-cell deterioration and associated loss in physiological insulin secretion is a central feature of T2D. β-cell function and/or mass progressively decline due to a sedentary lifestyle with abundant nutrient supply as a result of complex pathomechanisms eventually resulting in hyperglycemia and T2D [1]. Strategies to prevent β-cell failure or repair dysfunctional β-cell are urgently needed for a causative therapy targeting the cause of this severe metabolic disease.

Recently, we found the Pleckstrin homology domain leucine-rich repeat protein phosphatases 1 and 2 (PHLPP1, PHLPP2) highly upregulated in β-cells under a diabetic environment [2] and this directly correlates with β-cell death and impaired insulin secretion in human and rodent pancreatic β-cells in vitro and in vivo [2, 3]. PHLPP1/2 are members of the protein phosphatases metal-dependent (PPM) group of serine-threonine phosphatases (STPs) family and key mediators of pro-apoptotic signaling [4]. They directly dephosphorylate and inhibit pro-survival kinase AKT together with dephosphorylation and activation of pro-apoptotic MST1 [2, 4]; both pathways independently mediate β-cell failure [5, 6].

AKT serves as a main downstream executor of PI3K-IRS signaling; activated AKT boosts the mechanistic target of rapamycin complex 1 (mTORC1), which enhances growth and protein synthesis [7] and thus plays an instrumental role in the regulation of β-cell mass and function [6]. In contrast, MST1 activation results in excessive apoptosis and abolished insulin production, and is a strong mediator of diabetes progression [5]. Therefore, the PHLPP-AKT-MST1 triangle acts as a stress-sensitive survival pathway in β-cells and under physiological conditions, controls the fine balance of β-cell turnover. However, chronic overnutrition leads to sustained mTORC1 hyperactivation and subsequent PHLPP instigation which leads to imbalanced AKT/MST1 regulation and induction of β-cell apoptosis [2].

To substantiate the efficacy of genetic PHLPP1 inhibition seen in our recent study [2] in a severe in vivo mouse model of hyperglycemia and obesity-associated diabetes progression, we have deleted PHLPP1 in leptin receptor-deficient Lepr^db/db^ (db/db) mice. Db/db mice become severely obese leading to β-cell dysfunction and loss, chronic hyperglycemia, and diabetes by the age of 7–12 weeks. This was seen by the rise in glucose levels at the age of 6 weeks in db/db control mice with a heterozygous PHLPP1 deletion: db/db-PHLPP1^+/−^. In contrast, homozygous PHLPP1 deletion (db/db-PHLPP1^−^^/−^) led to drastically lower glucose levels to physiological conditions during the entire 6-week study period (Fig. 1A, B) [5, 8]. While db/db control mice showed severe glucose intolerance, it was significantly improved in the db/db-PHLPP1^−^^/−^ mice, together with a significant normalization of fasting glucose levels, indicating that PHLPP1 ablation prevents hyperglycemia (Fig. 1C, D). To test whether this metabolic improvement was due to changes in insulin sensitivity, we performed an intraperitoneal insulin tolerance test. After the insulin challenge, we did not see any differences in the ability to lower blood glucose levels in the two groups (Fig. 1E).

**Fig. 1:**
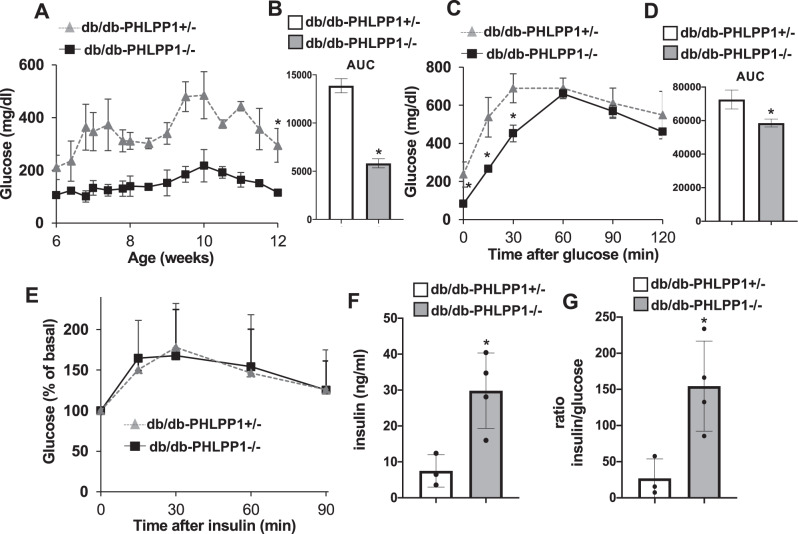
PHLPP1 depletion improves glycemia in the obese db/db mouse model of type 2 diabetes. Male db/db-PHLPP1^−^^/−^ (*n* = 4) and db/db-PHLPP1^+/−^ (*n* = 3) were monitored for **A**, **B** random fed blood glucose with respective area-under-the-curve (AUC) analysis from the age of 6 weeks throughout the 6-week experiment. **C**, **D** Intraperitoneal glucose tolerance test (ipGTT) with respective AUC analysis, **E** Intraperitoneal insulin tolerance test (ipITT) with basal glucose values normalized to 100%, **F** serum insulin, and **G** the ratio of serum insulin and blood glucose calculated at fed state at the end of the study in 12-weeks-old mice. Data show means ± SEM. *p < 0.05 db/db-PHLPP1^−^^/−^ compared to db/db-PHLPP1^+/−^; all by Student’s *t*-tests.

Despite the very low insulin sensitivity in db/db mice, the significantly elevated endogenous insulin production in db/db-PHLPP1^−^^/−^ mice with 4-fold increased serum insulin levels compared to controls (Fig. 1F) is a trigger for the improved glucose tolerance, which suggests that the anti-hyperglycemic effect seen in the PHLPP1^−^^/−^ mice likely comes from improved β-cell insulin secretion and/or mass. Indeed, the mice also showed a 5.7-fold increased insulin/glucose ratio at 12 weeks of age (Fig. 1G).

When we compared the heterozygous control db/db-PHLPP1^+/−^ mice to WT-db/db mice from different cohorts (e.g., refs. [8–10]), glucose and insulin levels were similar, which suggests that knocking out a single PHLPP1 allele has no effect on glucose metabolism. However, one needs to keep in mind that mice are from different studies and backcrosses.

To further investigate the mechanisms of the higher insulin production in db/db-PHLPP1^−^^/−^ mice, we dissected the pancreases and studied islet morphology and survival. Db/db-PHLPP1^−^^/^^−^ showed a 2-fold significantly increased insulin-positive β-cell area, which also reflected in a tendency to increased β-cell mass with generally much larger islets, compared to controls (Fig. 2A, B, D), while β-cell size remained similar in both PHLPP1^−^^/^^−^ and control mice (Fig. 2C). Such β-cell area restoration was due to an improved β-cell survival and enhanced β-cell replication. While β-cell apoptosis (shown by TUNEL-staining) was 4.7-fold reduced (Fig. 2E, F), β-cell proliferation (shown by Ki67-staining) was 8.7-fold increased (Fig. 2G, H) in db/db-PHLPP1^−^^/^^−^, compared to control. Islets from db/db-PHLPP1^+/^^−^ mice had fewer insulin-positive β-cells and an expansion of glucagon-positive α-cells, compared to db/db-PHLPP1^−^^/^^−^ mice (Fig. 2I–K), which were protected against such apparent α-cell hyperplasia. While they occurred very rare, we did not observe any significant changes in bi-hormonal insulin-glucagon double-positive cells between the two groups (Fig. 2L), ruling out the possibility of trans-differentiation from α- to β-cells in db/db-PHLPP1^−^^/^^−^ mice as the mechanism for the increased β-cell mass. This suggests a combined higher compensatory β-cell proliferation and a lower rate of apoptosis as a major mechanism of the β-cell mass restoration in db/db-PHLPP1^−^^/^^−^ mice.

**Fig. 2:**
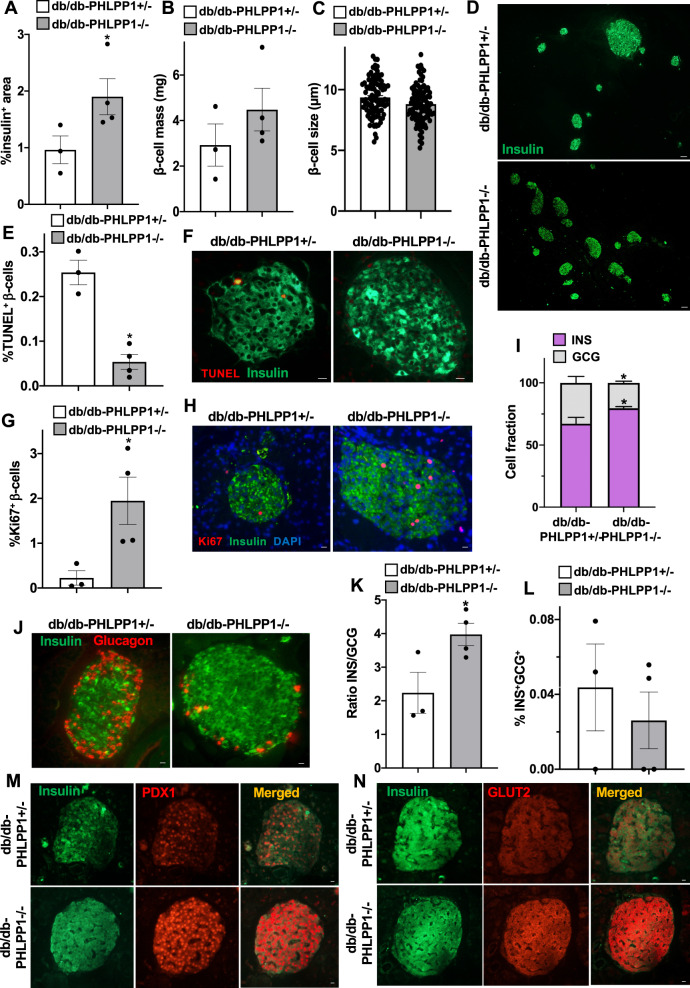
PHLPP1 depletion promotes β-cell survival in the obese db/db mouse model of type 2 diabetes. **A** Insulin-positive area and **B** β-cell mass analysis given as a mean percentage of the entire pancreatic section area from 10 sections/mouse throughout the whole pancreas, **C** the respective β-cell size analysis from 100 randomly chosen β-cells from each group and **D** representative insulin staining of the pancreas. **E**–**H** Quantitative analyses and representative images of **E**, **F** double-staining for TUNEL and **G**, **H** triple-staining for Ki67 both expressed as a percentage of insulin-positive β-cells (an average of 3827 and 3965 β-cells/ mouse were counted for TUNEL and Ki67 analyses, respectively for each group). Quantitative analyses (**I**, **K**) and representative images (**J**) of the percentage of glucagon-positive α-cells (red) and insulin-positive β-cells (green) together with quantitative analyses of insulin and glucagon colocalization (**L**; an average of 1455 β-cells and 529 α-cells/mouse were counted for each group). **M**, **N** Representative images for double-staining of **M** nuclear PDX1 and **N** GLUT2 expression. Data show means from all pooled analyses for each mouse ± SEM. *p < 0.05 db/db-PHLPP1^−^^/^^−^ vs. db/db-PHLPP1^+/^^−^ controls; all by Student’s *t*-tests. Scale bars depict 50 µm in **D** and 10 µm in **F**, **H**, **J**, **M**, **N**.

Metabolic disruption in protein networks led by chronic hyperglycemia and nutrient overload compromises the tightly regulated signaling pathways essential for β-cell identity, survival, and function. The transcription factor pancreatic duodenal homeobox-1 (PDX1) is indispensable for β-cell development and the control of glucose-stimulated insulin secretion in mature β-cells [11]. PDX1 level is declined in both human and rodent diabetic β-cells and this correlates with higher β-cell death and impaired insulin secretion [5, 12]. Correspondingly, db/db-PHLPP1^−^^/^^−^ mice expressed a much higher amount of PDX1 in the nucleus compared to the control db/db-PHLPP1^+/^^−^ counterpart (Fig. 2M). Our previous work identified PDX1 as a β-cell-specific MST1 substrate. Aberrant MST1 activity under diabetic condition promotes PDX1 degradation and inactivation through its direct phosphorylation [5]. Thus, PHLPPs as upstream activators of MST1 [2, 13] can indirectly regulate PDX1 degradation and thereby disrupt its function as a transcription factor [5]. Consequently, expression of the PDX1 downstream target GLUT2, the principal glucose transporter in rodent β-cells, which is essential for glucose sensing, was also increased in db/db-PHLPP1^−^^/^^−^ mice compared to heterozygous controls (Fig. 2N).

In addition, db/db-PHLPP1^−^^/^^−^ mice had a trend to lower body weight compared to the PHLPP1 heterozygous controls, however, this did not reach significance by the end of the study (data not shown) and did not lead to an improved insulin sensitivity; the anti-hyperglycemic effect of the PHLPP1 deletion seemed to have solely come from the improved β-cell phenotype.

Our findings demonstrate beneficial effects of PHLPP1 deficiency in severely diabetic db/db mice preventing hyperglycemia as well as improving β-cell survival and insulin production in vivo.

For the development of PHLPPs as a therapeutical target, the tumor-suppressing function of PHLPPs must not be underestimated, and such was tightly monitored in our studies. Neither PHLPP1^−^^/^^−^ nor db/db-PHLPP1^−^^/^^−^ mice developed anatomical or physiological defects. We did not detect any changes in organ growth nor tumorigenic abnormalities in PHLPP1^−^^/^^−^ mice [2], possibly due to the compensatory action of other PHLPP isoform PHLPP2 [14]. PHLPPs represent a promising target to further explore mechanisms underlying the pathophysiology of the broad signaling network in diabetic islets, which potentially help to rescue pancreatic β-cells in diabetes.

## Supplementary information

Supplementary Material

## Data Availability

All data needed to evaluate the conclusions in the paper are present in the paper and/or the Supplementary Materials. All raw data are available upon request from the authors.

## References

[1] Weir GC, Gaglia J, Bonner-Weir S (2020). Inadequate beta-cell mass is essential for the pathogenesis of type 2 diabetes. Lancet Diabetes Endocrinol.

[2] Lupse B, Annamalai K, Ibrahim H, Kaur S, Geravandi S, Sarma B (2021). Inhibition of PHLPP1/2 phosphatases rescues pancreatic beta-cells in diabetes. Cell Rep.

[3] Hribal ML, Mancuso E, Arcidiacono GP, Greco A, Musca D, Procopio T (2020). The phosphatase PHLPP2 plays a key role in the regulation of pancreatic beta-cell survival. Int J Endocrinol.

[4] Brognard J, Newton AC (2008). PHLiPPing the switch on Akt and protein kinase C signaling. Trends Endocrinol Metab.

[5] Ardestani A, Paroni F, Azizi Z, Kaur S, Khobragade V, Yuan T (2014). MST1 is a key regulator of beta cell apoptosis and dysfunction in diabetes. Nat Med.

[6] Dickson LM, Rhodes CJ (2004). Pancreatic beta-cell growth and survival in the onset of type 2 diabetes: a role for protein kinase B in the Akt?. Am J Physiol Endocrinol Metab.

[7] Saxton RA, Sabatini DM (2017). mTOR signaling in growth, metabolism, and disease. Cell.

[8] Ardestani A, Li S, Annamalai K, Lupse B, Geravandi S, Dobrowolski A (2019). Neratinib protects pancreatic beta cells in diabetes. Nat Commun.

[9] Dhanesha N, Joharapurkar A, Shah G, Kshirsagar S, Patel V, Patel K (2013). Treatment with exendin-4 improves the antidiabetic efficacy and reverses hepatic steatosis in glucokinase activator treated db/db mice. Eur J Pharmacol.

[10] Bojar D, Scheller L, Hamri GC, Xie M, Fussenegger M (2018). Caffeine-inducible gene switches controlling experimental diabetes. Nat Commun.

[11] Johnson JD, Ahmed NT, Luciani DS, Han Z, Tran H, Fujita J (2003). Increased islet apoptosis in Pdx1+/- mice. J Clin Invest.

[12] Guo S, Dai C, Guo M, Taylor B, Harmon JS, Sander M (2013). Inactivation of specific beta cell transcription factors in type 2 diabetes. J Clin Invest.

[13] Qiao M, Wang Y, Xu X, Lu J, Dong Y, Tao W (2010). Mst1 is an interacting protein that mediates PHLPPs’ induced apoptosis. Mol Cell.

[14] Chen M, Pratt CP, Zeeman ME, Schultz N, Taylor BS, O’Neill A (2011). Identification of PHLPP1 as a tumor suppressor reveals the role of feedback activation in PTEN-mutant prostate cancer progression. Cancer Cell.

