# Supplementary material for: PHLPP1 deletion restores pancreatic β-cell survival and normoglycemia in the db/db mouse model of obesity-associated diabetes
Source: Cell Death Discov. 2022 Feb 8;8:57. doi: 10.1038/s41420-022-00853-5 (PMC8825859; doi:10.1038/s41420-022-00853-5)
Supplement: Supplementary file 1 — Supplementary Material [file 41420_2022_853_MOESM1_ESM.docx]

**Supplementary Material**

**Methods**

**Animals**

Heterozygous leptin receptor deficient mice on the C57BLKS/J background were purchased from Jackson Laboratory, ME, USA; their interbreeding obtained diabetic Lepr_db/db_ (db/db) as well as non-diabetic heterozygous Lepr^db/+^ (db/+) littermates. To generate db/db-PHLPP1^-/-^ mice, PHLPP1^-/-^ mice (RRID:MGI:5795609; generously provided by Alexandra Newton, UCSD) on a C57BL/6J genetic background were backcrossed for 7 generations with heterozygous db/+ mice (to reach more than 99% of C57BLKS/J background). PHLPP1-KO mice have been fully characterized in our previous study by western blotting in both isolated islets and isolated Mouse Primary Embryonic Fibroblasts (MEFs) to ensure PHLPP1 genetic absence (1).

db/+-PHLPP1^+/-^ and db/+-PHLPP1^-/-^ mice were then intercrossed to generate db/db-PHLPP1^-/-^ and their corresponding control db/db-PHLPP1^+/-^. For both groups, blood was obtained from the tail vein of nonfasted mice, and glucose was measured using a Glucometer (FreeStyle; Abbott, IL, USA). Mice were set up for the experiment at the age of 6 weeks and sacrificed at the age of 12 weeks. All mice were fed with normal diet (ND, Harlan Teklad Rodent Diet 8604, containing 12.2, 57.6 and 30.2% calories from fat, carbohydrate and protein, respectively) and housed in a temperature-controlled room with a 12 h light-dark cycle and were allowed free access to food and water in agreement with NIH animal care guidelines, §8 German animal protection law, German animal welfare legislation and with the guidelines of the Society of Laboratory Animals (GV-SOLAS) and the Federation of Laboratory Animal Science Associations (FELASA). Genotyping of the mice for the *db* mutation and *PHLPP*-deletion was done with following primers: PHLPP-forward TAGGAGAGACTAGTGACATC; PHLPP-reverse TGAGCTTATACGCTGTGATGC; PHLPP-KO64 TCAAAGTGGGAAAGGAAGGA; Db-common GCTGCAGAATGGACGGTTGA; Db-WT GCAGTGCACAGGCTCAGGAA; Db-mutant AGCCACTACAATCCACCCCTTG.

**Glucose and insulin tolerance tests and insulin secretion**

For intraperitoneal glucose tolerant tests (ipGTT), mice were fasted overnight for 12h and injected i.p. with glucose (B.Braun, Germany) at a dose of 1g/kg body weight. Blood samples were collected at time points 0, 15, 30, 60, 90, and 120 min for glucose measurements by using a Glucometer (FreeStyle; Abbott, IL, USA). For i.p. insulin tolerance tests (ipITT), mice were initially fasted for a period of 4h followed by recombinant human insulin injection (Novo Nordisk, Denmark) at a dose of 0.75 U/kg body weight. Glucose concentration was determined with the Glucometer at time points 0, 15, 30, 60 and 90 min. Blood samples for insulin secretion was collected before (0 min) and after (15 and 30 min) i.p. injection of glucose (2g/kg body weight) and measured by using ultrasensitive mouse ELISA kit (ALPCO Diagnostics, NH, USA).

**Immunohistochemistry**Mouse pancreases were dissected and fixed in 4% formaldehyde at 4°C for 8h and dehydrated before embedding in paraffin. Mouse sections (2 μm) were deparaffinized, rehydrated and incubated overnight at 4°C with rabbit anti-Ki-67 (#M7249; Dako), rabbit anti-PDX1 antibody (#47267; Abcam, UK), mouse anti-glucagon antibody (#10988; Abcam, UK), rabbit anti-GLUT2 antibody (#07-1402; Chemicon, CA, USA) or for 2 h at room temperature with anti-insulin (#A0546; Dako), followed by fluorescein isothiocyanate (FITC)- or Cy3-conjugated secondary antibodies (Jackson ImmunoResearch Laboratories, PA, USA). Slides were mounted with Vectashield with 4′6-diamidino-2-phenylindole (DAPI) (Vector Labs, CA, USA). Pancreatic β-cell apoptosis was analyzed by the terminal deoxynucleotidyl transferase-mediated dUTP nick-end labeling (TUNEL) technique according to the manufacturer’s instructions (In situ Cell Death Detection Kit, TMR red; Roche, Switzerland) and double stained for insulin. Fluorescence was analyzed using a Nikon MEA53200 (Nikon GmbH, Germany) microscope, and images were acquired using NIS-Elements software from Nikon.

**Morphometric analysis**

For morphometric data, ten sections (spanning the width of the pancreas) per mouse were analyzed. Insulin-positive area and β-cell mass were determined by computer-assisted measurements by using a Nikon MEA53200 (Nikon GmbH, Germany) microscope, and images were acquired by using NIS-Elements software from Nikon. Mean percent β-cell fraction per pancreas was calculated as the ratio of insulin-positive and whole pancreatic tissue area (2).

**Statistical analyses**

At least three mice as independent biological replica (referred to “n”) were used as reported in the figure legend. Data are presented as means ± SEM. Mean differences were determined by Student’s t- tests. P value <0.05 was considered statistically significant.

**Study approval**

Ethical approval for the mouse experiments had been granted by the Bremen Senate (Senator for Science, Health and consumer protection) and we have complied with all relevant ethical regulations for animal testing and research.

1. Lupse B, Annamalai K, Ibrahim H, Kaur S, Geravandi S, Sarma B, et al. Inhibition of PHLPP1/2 phosphatases rescues pancreatic beta-cells in diabetes. Cell Rep. 2021;36(5):109490.

2. Ardestani A, Paroni F, Azizi Z, Kaur S, Khobragade V, Yuan T, et al. MST1 is a key regulator of beta cell apoptosis and dysfunction in diabetes. Nat Med. 2014;20(4):385-97.
